# Synaptic mechanisms underlying the elevated sympathetic outflow in fructose-induced hypertension

**DOI:** 10.3389/fphys.2024.1365594

**Published:** 2024-03-04

**Authors:** Yun Zhu, Haiying Sun, Hongjie Wang, Na Li

**Affiliations:** ^1^ Department of Otorhinolaryngology, Union Hospital, Tongji Medical College, Huazhong University of Science and Technology, Wuhan, China; ^2^ Department of Anesthesiology, Affiliated Hospital of Hebei University, Baoding, China; ^3^ Department of Physiology, School of Basic Medical Sciences, Hebei University, Baoding, China

**Keywords:** metabolic syndrome, nitric oxide, sympathetic outflow, hypertension, nucleus tractus solitarius neurons, glutamate synaptic inputs

## Abstract

Metabolic syndrome is associated with cardiovascular dysfunction, including elevated sympathetic outflow. However, the underlying brain mechanisms are unclear. The nucleus tractus solitarius (NTS) critically regulates autonomic reflexes related to cardiovascular function and contains neurons projecting to the caudal ventrolateral medulla (CVLM). Nitric oxide (NO) is a diffusible free-radical messenger in the vascular, immune, and nervous systems. In this study, we determine if NO in the NTS is involved in the synaptic plasticity underlying the elevated sympathetic outflow in fructose-induced hypertension. We retrogradely labeled CVLM-projecting NTS neurons through the injection of FluoSpheres into the CVLM in a fructose-fed rat model to determine the cellular mechanism involved in increased sympathetic outflow. Fructose feeding increased the blood pressure and glucose levels, which represent metabolic syndrome. We found that fructose feeding reduces the NO precursor L-arginine-induced increase in the firing activity of CVLM-projecting NTS neurons. Furthermore, fructose feeding reduces the L-arginine-induced increase in presynaptic spontaneous glutamatergic synaptic inputs to NTS neurons, while NO donor DEA/NO produces an increase in glutamatergic synaptic inputs in fructose-fed rats similar to that in vehicle-treated rats. In addition, fructose feeding reduces the NO-induced depressor response and sympathoinhibition. These data suggested that fructose feeding reduced NO production and, thus, the subsequent NO-induced glutamate releases in the NTS and depressor response. The findings of this study provide new insights into the central mechanisms involved in the neural control of cardiovascular and autonomic functions in the NTS in metabolic syndrome.

## 1 Introduction

Metabolic syndrome is associated with increased cardiovascular morbidity and mortality and is becoming an increasingly prevalent clinical problem (Alberti et al., 2006; Kassi et al., 2011). Metabolic syndrome not only confers an adverse prognosis in the general population but also in patients with hypertension, diabetes, and hypercholesterolemia, in whom it further increases the already elevated cardiovascular risk (Malik et al., 2004; Mancia et al., 2007). Furthermore, people with metabolic syndrome have a higher probability of developing hypertension or diabetes (Lloyd-Jones et al., 2004; Hanley et al., 2005; Wilson et al., 2005). An animal model is the fructose-induced hypertensive rat with hyperglycemia, hypertriglyceridemia, insulin resistance, and moderate hypertension as a result of being fed a fructose-rich diet. The metabolic changes in fructose-fed rats resemble human metabolic syndrome, and thus, fructose-fed rats have been used to study the relationship between metabolic defects and autonomic function without confounding factors, such as severe hypertension, obesity, diabetes, or genetic contributions (Hwang et al., 1987; Abdelrahman et al., 2018).

Located within the dorsomedial medulla oblongata, the nucleus tractus solitarius (NTS) plays an important role in regulating autonomic reflexes related to cardiovascular function (MacDonald and Ellacott, 2020). The stimulation of the NTS with L-glutamate produces a decrease in the sympathetic outflow and blood pressure, an effect mimicking the baroreflex (Talman et al., 1980). On the other hand, electrolytic lesions of the NTS cause the elevation of the sympathetic vasomotor tone and the loss of the baroreflex control of blood pressure and heart rate in both humans (Montgomery, 1961) and animals (Doba and Reis, 1973). The NTS is a heterogeneous nucleus and has reciprocal connections with various other regions, including the rostral ventrolateral medulla (RVLM), caudal ventrolateral medulla (CVLM), paraventricular nucleus of the hypothalamus, and other forebrain nuclei (Ricardo and Koh, 1978; Stornetta et al., 2016). The NTS is the primary central site receiving afferents of the baroreflex (Elsaafien et al., 2022). Projection from the NTS to the CVLM is an important pathway mediating the cardiovascular baroreflex (Dampney et al., 2003).

Nitric oxide (NO) is a diffusible free radical that has been recognized as a biological messenger in the vascular, immune, and nervous systems. NO is liberated during the conversion of L-arginine into citrulline, a process that is catalyzed by endothelial nitric oxide synthase (eNOS), neuronal NOS (nNOS), and inducible NOS (Palmer et al., 1988). NO regulates the cardiovascular function not only through direct vasodilatation but also through action in the central nervous system (CNS). Both eNOS and nNOS are present in the cell soma and nerve terminals in the NTS (Vincent and Kimura, 1992; Ruggiero et al., 1996; Takagishi et al., 2014). NO plays an important role in the regulation of the sympathetic nerve activity, arterial blood pressure, and heart rate in the NTS (Tseng et al., 1996; Li et al., 2019; Kishi, 2023) through distinct signal transduction pathways (Stamler et al., 1997; Ahern et al., 2002), including S-nitrosylation on the receptor protein (Nakamura and Lipton, 2016; Sharma et al., 2023), formation of peroxynitrite (Trabace and Kendrick, 2000), and the activation of the soluble isoform of guanylyl cyclase (sGC) and the subsequent elevation of the intracellular concentration of 3′,5′-cyclic guanosine monophosphate (cGMP) (Garthwaite, 2016; Peters et al., 2018). Glutamate is the predominant neurotransmitter involved in processing the visceral afferent information in the NTS (Bonham and Chen, 2002; Ohi et al., 2014). Glutamate exists in the vagal afferent terminals in the NTS, and the stimulation of the baroreceptor afferent causes the release of glutamate in the NTS (Lawrence and Jarrott, 1994; Ohta et al., 1996). Glutamate stimulates two superfamilies of receptors: metabotropic and ionotropic receptors. The metabotropic glutamate receptors are G-protein-coupled receptors; the ionotropic glutamate receptors are ligand-gated receptor channels that have been named according to their most specific agonists, α-amino-3-hydroxy-4-isoxazolepropionic acid (AMPA), kainic acid, and N-methyl-D-aspirate (NMDA), which contribute to the full expression of various autonomic reflexes, such as the baroreflex and cardiopulmonary reflexes (Ohi et al., 2014; Li et al., 2019; Neyens et al., 2020). NO may regulate neuronal activity by affecting both the inhibitory and excitatory neurotransmitter release in the NTS. NO plays an important role in the regulation of glutamate release in the NTS since the NO donor increases the extracellular glutamate level, and the NOS inhibitor N^G^-monomethyl-L-arginine reduces the basal level of glutamate in the NTS (Lin H. C. et al., 2000). Furthermore, the NO donor, diethylammonium (Z)-1-(N,N-diethylamino)diazen-1-ium-1,2-diolate (DEA/NO), increases the tractus solitarius (TS)-evoked glutamatergic excitatory postsynaptic potentials in the NTS (Wang et al., 2007). In this study, we used a NO precursor and donor to determine the role of NO in regulating glutamatergic synaptic inputs and the elevated sympathetic outflow in fructose-induced hypertension.

## 2 Materials and methods

### 2.1 Fructose-induced hypertensive rats

Male Sprague–Dawley (SD) rats (240–280 g) were provided by the Experimental Animal Center of Hebei Medical University. The animal use protocol listed below was reviewed and approved by the Hebei University Animal Ethics and Welfare Committee (AEWC), and the AEWC also approved the protocol no. IACUC-2019002SR. The rats were housed in clean cages with a constant temperature (25°C) and photoperiod (12-h light/dark cycle). All experimental procedures were conducted according to the guidelines of the Animal Care and Ethics Committee of Hebei University, China. All of the rats were housed under controlled environmental conditions with a standard rodent diet (Harlan) available *ad libitum*. The rats were randomly divided into two groups—the vehicle-treated group (*n* = 8) was given regular tap water to drink for 6 weeks, and the fructose-fed group (*n* = 8) was given a fructose solution (10% w/v) to drink for 6 weeks (Mayer et al., 2006; Mayer et al., 2008). Blood pressure was measured in each rat using a noninvasive tail-cuff system, and their plasma glucose levels were measured using a glucose assay kit.

### 2.2 Retrograde labeling of CVLM-projecting NTS neurons

**FIGURE 1:**
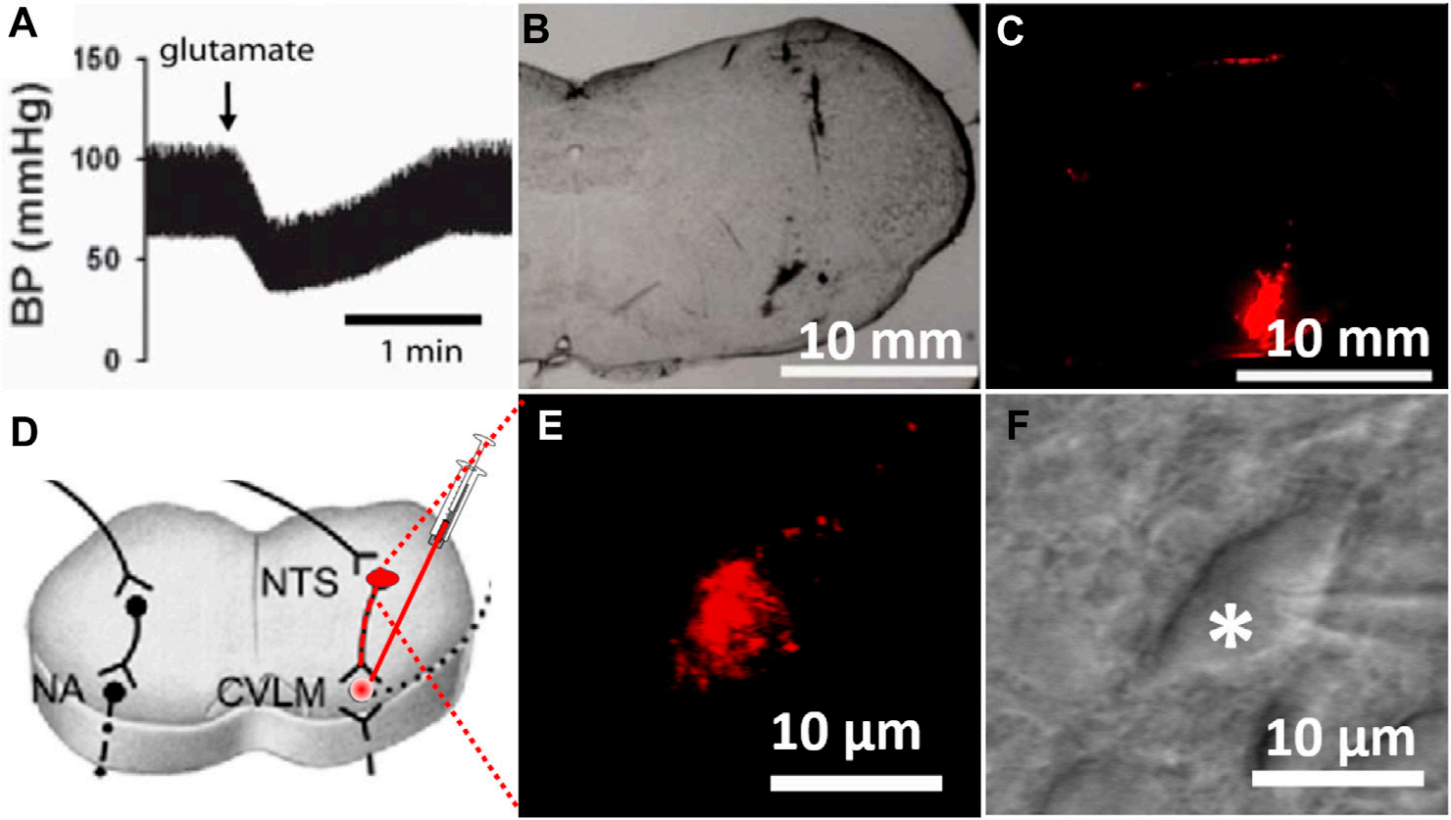
Labeling of CVLM-projecting NTS neurons. **(A)** Depressor response to the microinjection of glutamate (100 mM; 20 nL) into the CVLM. **(B,C)** Photomicrographs of brainstem slices viewed under a light microscope and fluorescent illumination at the CVLM level (bregma, 14.0 mm) with FluoSpheres microinjected into the CVLM (red). **(D)** Diagram illustrating retrogradely labeling NTS neurons by injection of tracer into the CVLM. **(E,F)** FluoSpheres-labeled NTS neuron (red) in a slice viewed with fluorescence illumination **(E)** and differential interference contrast optics (F, indicated by *) (magnification: ×60). BP, blood pressure; CVLM, caudal ventrolateral medulla; NTS, nucleus tractus solitarius; NA, nucleus ambiguus.

The identification of the CVLM and the injection of FluoSpheres into the CVLM were carried out as described previously (Li and Yang, 2007). Briefly, following the induction of anesthesia with a mixture of ketamine (50 mg/kg) and xylazine (8 mg/kg) injected into the thigh muscles, a catheter was inserted into the right femoral artery to monitor the arterial blood pressure. The rat head was mounted on a stereotaxic apparatus. Parietal–occipital craniotomy was performed to expose the dorsal surface of the medulla oblongata through the retraction of the atlanto-occipital membrane, and the CVLM was targeted at the following coordinates: 0.5 mm rostral to the calamus scriptorius (defined as the caudalmost tip of the area postrema), 1.6–1.8 mm lateral to the midline, and 1.8–2.1 mm deep into the dorsal surface of the brainstem (Hermes et al., 2006). CVLM localization was verified by observing depressor responses to microinjections of glutamate (100 mM; 20 nL) (Li and Yang, 2007). The CVLM was considered correctly localized when glutamate injections decrease the arterial blood pressure by at least 20 mmHg. After the identification of the CVLM, a rhodamine-labeled fluorescent microsphere suspension (0.04 µm, FluoSpheres, Invitrogen) was bilaterally injected into the CVLM using an injector (Nanoject II) and a glass micropipette (OD: 1.0; ID: 0.5 mm), pulled using a micropipette puller (Model P-97, Sutter), and subsequently ground so that they have a beveled tip with an outer diameter of 20–30 µm. After the injection of FluoSpheres, the muscles were sutured to close the wound. The brainstem slices (300 µm in thickness) were sectioned 1 week after the FluoSpheres injection, as described in a previous study (Li and Yang, 2007). To verify that FluoSpheres are injected into the CVLM, brainstem slices were examined histologically after the rats were euthanized (Figure 1C). Figure 1 depicts the microinjection of FluoSpheres into the CVLM (A–C) and the identification of labeled CVLM-projecting NTS neurons in perfused brainstem slices as observed under an upright microscope with a combination of epifluorescence illumination and infrared light and differential interference contrast optics (D–E).

### 2.3 *In vitro* electrophysiological experiments

Following the microinjection of FluoSpheres into the CVLM, we cut the rat brainstem slices. Briefly, the rats were anesthetized with isoflurane (2% in O_2_) and decapitated. The brainstem was quickly removed and placed in ice-cold artificial cerebrospinal fluid (aCSF) containing 125 mM NaCl, 3 mM KCl, 1.25 mM NaH_2_PO_4_, 2.4 mM CaCl_2_, 1 mM MgCl_2_, 10 mM glucose, and 25 mM NaHCO_3_ saturated with 95% O_2_ and 5% CO_2_. A tissue block containing the NTS was dissected and affixed with glue to the cutting stage of a vibratome. Four to six coronal slices were sectioned at 250–300-µm thicknesses in the ice-cold aCSF. The brainstem slices were placed in a glass-bottomed chamber and fixed with a grid of parallel nylon threads supported by a U-shaped stainless-steel weight. The brainstem slices were visualized using an upright microscope (BX50WI, Olympus) with a combination of epifluorescence illumination and infrared light and differential interference contrast (IR-DIC) optics. The FluoSpheres-labeled NTS neurons (red) were briefly identified by epifluorescence illumination and used for electrophysiological experiments.

The recording pipettes were triple-pulled using borosilicate capillaries (OD: 1.2 mm; ID: 0.86 mm) using a puller (P-97, Sutter). The tips of the pipettes were fire-polished to a final shape using a microforge. The resistance of the recording pipette was 3–6 MΩ when the pipette was filled with a solution containing 130 mM potassium gluconate, 1 mM MgCl_2_, 10 mM HEPES, 10 mM EGTA, 1 mM CaCl_2_, and 4 mM ATP-Mg, adjusted to pH 7.25 with 1 M KOH (osmolarity 290–300 mOsm). All the recordings were corrected for the liquid junction potential, which was defined as the difference in the voltage between pipettes filled with the recorded pipette internal solution and external perfusion solution. Each brainstem slice was perfused at 3 mL/min at 34°C maintained using an in-line solution heater and temperature controller. It took approximately 1 min to completely exchange the solution in the recording chamber at a perfusion rate of 3 mL/min. Whole-cell configuration in NTS neurons was established under visual control using an upright microscope with a combination of epifluorescence illumination and IR-DIC optics. A tight GΩ seal was subsequently obtained between the recording pipette and the cell membrane of the labeled NTS neuron. The cell membrane was then ruptured by the application of a negative pressure pulse to the pipette. Recordings began 5 min after whole-cell access was established and had reached a steady state. Electronic signals were processed using a MultiClamp 700B amplifier. The signals were filtered at 1–2 kHz, digitized at 10 kHz using Digidata 1440, and saved to the hard drive of a computer. The membrane voltage and spontaneous action potentials were recorded in the current-clamp mode.

The evoked excitatory postsynaptic currents (eEPSCs) in the labeled NTS neurons were recorded in the presence of 1 μM gabazine and 5 μM strychnine at a holding potential of −70 mV. We evoke synaptic currents through the electrical stimulation of the TS ipsilateral to the recording site through a bipolar tungsten electrode connected to a stimulator. The stimulation was limited to 10-V, 0.1-ms square-wave pulses to minimize the voltage spread outside the TS. The stimuli were delivered through a high-impedance (10 MΩ) bipolar tungsten electrode (1-µm tips separated by 80 µm) at a frequency of 0.2 Hz. The mean distance between the stimulating electrode and the recorded neuron was maintained at 200–500 µm. Monosynaptic postsynaptic currents were identified on the basis of short-onset latencies (≤4.5 ms), variation in the latencies (<0.5 ms) of eEPSCs, and the absence of the conduction failure of eEPSCs in response to a 20-Hz electrical stimulation (Berry and Pentreath, 1976; Aylwin et al., 1997). The evoked inhibitory postsynaptic currents (eIPSCs) were recorded in the presence of 20 µM 6-cyano-7-nitroquinoxaline-2,3-dione (CNQX, a glutamate non-NMDA receptor antagonist) and 50 µM DL-2-amino-5-phosphonopentanoic acid (AP5, an NMDA receptor antagonist) at a holding potential of 0 mV. We elicited eIPSCs by both TS stimulation and focal stimulation. For focal stimulation, the tip of the stimulation electrode was placed close to the recorded neurons. A sodium channel blocker, N-ethyl bromide quaternary salt (QX-314, 5 mM), was included in the recording pipette solution to block the sodium currents and possible postsynaptic effects in these voltage-clamp experiments. The spontaneous and miniature EPSCs were recorded in the presence of 1 µM gabazine at a holding potential of −70 mV, and the spontaneous and miniature IPSCs were recorded in the presence of 20 µM CNQX, 50 µM AP5, and 5 μM strychnine at a holding potential of 0 mV. Tetrodotoxin (1 µM) was added to the perfusion solution for the recording of miniature IPSCs and EPSCs. All the drugs were prepared immediately before the experiments and applied to the recording chamber using syringe pumps. The calculated high-concentration drugs were delivered using pumps and mixed with the main perfusion flow in the final concentration before their entry into the recording chamber.

### 2.4 *In vivo* recordings of hemodynamics and sympathetic nerve activity and microinjection

The rats were initially anesthetized using 2% isoflurane in O_2_, and isoflurane was discontinued after a mixture of α-chloralose (60–75 mg/kg) and urethane (800 mg/kg) was intraperitoneally administered. Adequate anesthesia was confirmed by the absence of the withdrawal response to a noxious stimulus (tail pinch). The trachea was cannulated, and the rats were mechanically ventilated using a rodent ventilator (Model 683, Harvard Apparatus, South Natick, MA). The left femoral artery was cannulated, and the arterial blood pressure was measured using a pressure transducer (PT300, Grass Instruments, Quincy, MA). The left jugular vein was cannulated for the intravenous injection of drugs. The left kidney was exposed through a left flank incision via a retroperitoneal approach (Li et al., 2001). A small branch of the renal nerve was isolated and carefully dissected from the renal vasculature and the surrounding tissue using an operating microscope. The renal nerve was cut distally to ensure that afferent activity was not recorded. The renal nerve was then bathed in mineral oil and placed on a stainless-steel recording electrode. The nerve signal was amplified (×20,000–30,000) and the bandpass was filtered (100–3,000 Hz) using an alternating current amplifier (Model P511, Grass Instruments). The renal sympathetic nerve activity (RSNA) was monitored using an audio amplifier (Model AM10, Grass Instruments). The RSNA and arterial blood pressure were recorded using a 1401-Plus analog-to-digital converter and the Spike2 system (Cambridge Electronic Design, Cambridge, United Kingdom) displayed and stored on a Pentium computer. The heart rate was measured by triggering the blood pressure pulse. The background noise was determined after the rats were euthanized by an overdose of sodium pentobarbital at the end of each experiment. Background noise levels were subtracted from the nerve activity, and the percentage change in the RSNA from the baseline value was also calculated.

For the NTS microinjection, the dorsal surface of the medulla was surgically exposed through the atlanto-occipital membrane. A glass microinjection pipette (tip diameter, 20–30 µm) was advanced to the NTS according to the following coordinates: 0.5 mm rostral to the calamus, ±0.5 mm lateral to the midline, and 0.5 mm ventral from the surface of the medulla (Dias et al., 2005). The drug was injected using a calibrated microinjection system under an operating microscope. After drug microinjection, the glass pipette was left in place for 1–2 min to ensure the adequate delivery of the drug to the injection site. The micropipette was withdrawn and placed at the respective stereotaxic coordinates for injection into the contralateral NTS. The accurate microinjection site and the spread of the injected drug within the NTS were verified histologically. The ejection volumes were measured by monitoring the movement of the fluid meniscus in a pipette barrel using a 150× compound microscope equipped with a fine reticule. At the end of the experiment, 50 nL of 2% Chicago Blue dye or 1% fluorescent dye was ejected from the same microinjection pipette at the same site. After euthanasia, the brainstem was removed rapidly and fixed in 10% buffered formalin. Frozen 40-µm coronal sections were cut using a freezing microtome, and the dye spots and spread area were identified to confirm their locations in the NTS according to the atlas of Paxinos and Watson. Data were excluded if the microinjection site and drug spread were not located within the NTS.

### 2.5 Data analysis

For the *in vitro* slice data, the firing activity was analyzed off-line using the peak detection program Mini Analysis 6.0 (Synaptosoft Inc.). The firing rate was obtained by averaging the frequency and membrane potentials over a period of 3–6 min before, during, and after drug application. A neuron was considered responsive if its firing activity was altered by more than 20% following the drug application. The junction potential was corrected off-line on the basis of the composition of the internal and external solutions used for recordings. The spontaneous and miniature EPSCs were detected by the rapid rise time of the signal over an amplitude threshold set above the background noise. At least 100 randomly selected spontaneous or miniature EPSCs were used in each analysis. The amplitude of the eEPSCs was analyzed using Clampfit 9.0 software. Statistical data were presented as the mean ± S.E. The effects of drugs on the firing activity, membrane potentials, and spontaneous and miniature EPSCs and eEPSCs were determined using the Wilcoxon signed-rank test or non-parametric analysis of variance (ANOVA) (Kruskal–Wallis) with Dunn’s *post hoc* test. *p* <0.05 was considered to be statistically significant.

The *in vivo* data on the RSNA, arterial blood pressure, and heart rate were analyzed using Spike2 software (Cambridge Electronic Design). The mean arterial blood pressure was derived from the arterial blood pressure and calculated as the diastolic pressure plus one-third of the pulse pressure. The RSNA signals were rectified, and background noise was subtracted using the level obtained after the rats were euthanized by sodium pentobarbital overdose. The nerve signal was integrated with a 1-s time constant. Control values were obtained from the average during the 60-s period immediately before each injection. The RSNA was presented as the percentage change from baseline activity since the baseline RSNA varies from animal to animal. The arterial blood pressure, heart rate, and RSNA values, following each intervention, were calculated by averaging the parameters over 30 s when the maximal responses occurred. To compare the differences in the responses of the arterial blood pressure, heart rate, and the RSNA and HR within the experimental groups, repeated-measures ANOVA with Dunnett’s *post hoc* test was performed. A two-way ANOVA with Bonferroni’s *post hoc* test was used to compare both the raw data and the relative changes of the parameters between experimental groups. *p* <0.05 was considered statistically significant.

## 3 Results

Fructose-fed rats had a significantly higher systolic blood pressure and plasma glucose levels than the vehicle-treated rats (Table 1). The mean body weight did not differ between the vehicle- and fructose-fed groups.

**TABLE 1:** Body weight, systolic blood pressure, and plasma glucose level in fructose-fed and vehicle-treated rats.

Experimental group	Body weight, g	Systolic blood pressure, mmHg	Plasma glucose level, mg dl^-1^
Vehicle-treated rats (*n* = 8)	354.1 ± 5.5	110 ± 2	133.3 ± 4.8
Fructose-fed rats (*n* = 8)	360.7 ± 6.0	153 ± 3^a^	152.0 ± 5.2^a^

^a^ *p* <0.05 compared with the vehicle-treated group using unpaired Student’s t-test.

### 3.1 Fructose feeding reduces the NO-induced excitatory effect on NTS neurons

**FIGURE 2:**
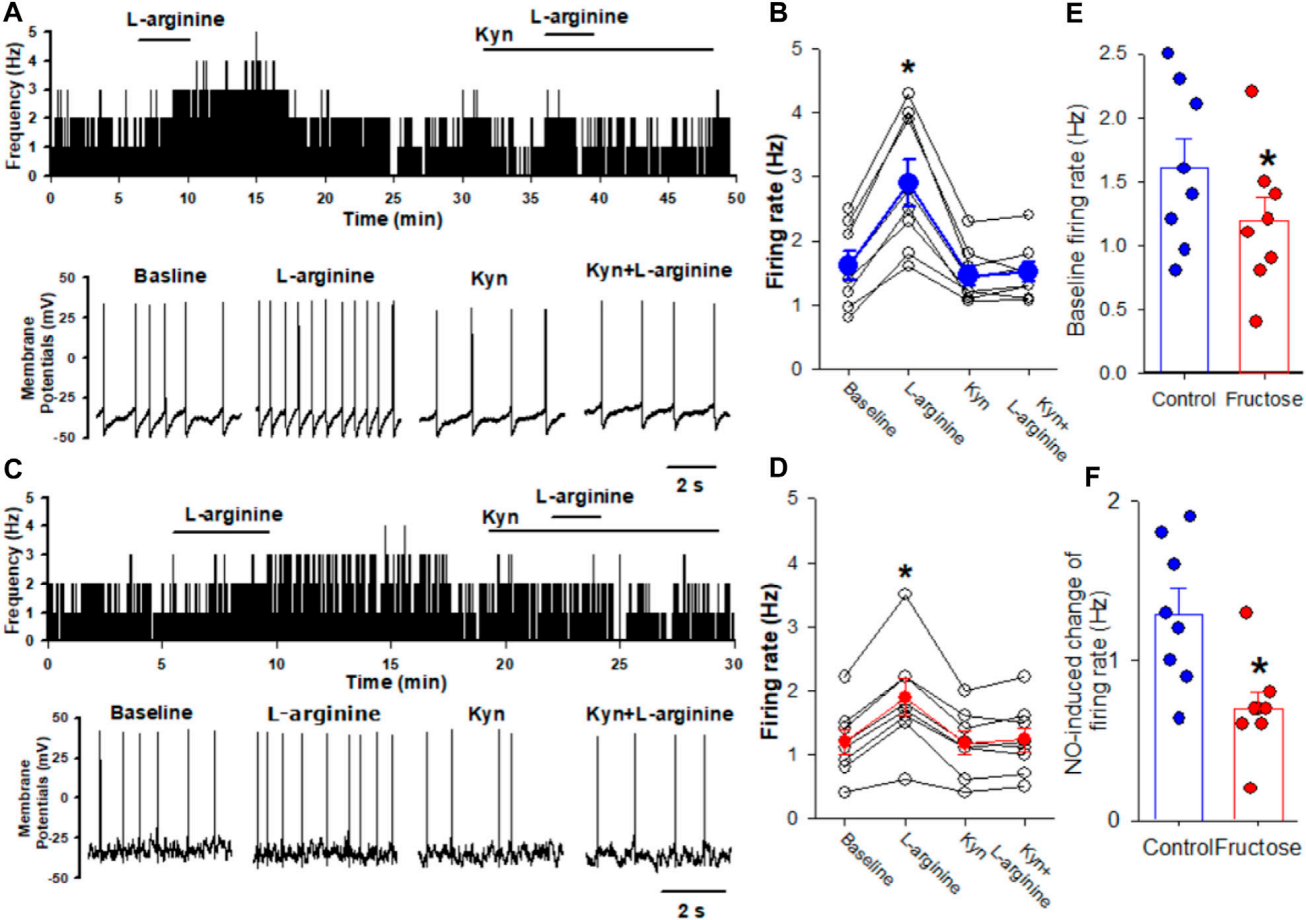
Effects of L-arginine on the firing activity of labeled CVLM-projecting NTS neurons in fructose-fed rats. **(A,B)** Representative tracings **(A)** and summary data **(B)** show the effect of 100 μM L-arginine on the firing activity of labeled NTS neurons in vehicle-treated rats. Note that blocking glutamate receptors with kynurenic acid (Kyn) abolished L-arginine-induced increases in the firing activity. **(C,D)** Representative tracings **(C)** and summary data **(D)** show the effect of 100 μM L-arginine on the firing activity of labeled NTS neurons in fructose-fed rats. **(E)** The baseline firing rate of NTS neurons in fructose-fed rats was lower than that in vehicle-treated rats. **(F)** L-arginine induced a reduced excitatory effect on the firing activity of the labeled NTS neurons in fructose-fed rats compared to vehicle-treated rats. Data are presented as the mean ± SEM (*p <0.05 compared with the baseline value; Kruskal–Wallis ANOVA, followed by Dunn’s *post hoc* test). Kyn, kynurenic acid.

Previous studies have shown that blocking ionotropic glutamate receptors attenuates the depressor effect elicited by the microinjection of a NO donor into the NTS (Lo et al., 1997; Lin et al., 1999). Thus, we determined the effect of NO on the firing activity of the NTS neurons projecting to the CVLM in fructose-fed rats. The CVLM-projecting NTS neurons were retrogradely labeled by the injection of FluoSpheres into the CVLM (Figure 1). We then determined the role of synaptic glutamate release in the effect of NO-labeled NTS neurons. We found that the NO donor L-arginine (100 μM) increased spontaneous firing activity from 1.6 ± 0.2 to 2.9 ± 0.4 Hz in 8 labeled NTS neurons, with a depolarization of membrane potentials from −53.8 ± 3.3 to −49.7 ± 3.2 mV. The concentration of L-arginine was chosen from a previous study (Tagawa et al., 1994). To determine the role of glutamatergic synaptic inputs in the L-arginine-induced increase in the firing activity of these labeled neurons, the ionotropic glutamate receptor antagonist, kynurenic acid (2 mM), was bath-applied. Kynurenic acid alone decreased the firing rate from 1.6 ± 0.2 to 1.4 ± 0.2 Hz in 8 labeled NTS neurons. The subsequent application of 100 μM L-arginine did not alter the firing activity in the presence of 2 mM kynurenic acid (Figure 2). The bath application of 100 μM L-arginine had a lesser excitatory effect on the firing activity of the labeled NTS neurons (*n* = 8) in fructose-fed rats than that in vehicle-treated rats (Figure 2). Furthermore, the baseline firing rate of these CVLM-projecting NTS neurons was significantly lower in fructose-treated rats than that in vehicle-treated rats. These data provided substantial evidence that the excitatory effect of endogenous NO on autonomic NTS neurons was reduced in fructose-fed rats.

### 3.2 Fructose feeding reduces the NO-induced increase in presynaptic spontaneous glutamatergic-synaptic inputs to NTS neurons

**FIGURE 3:**
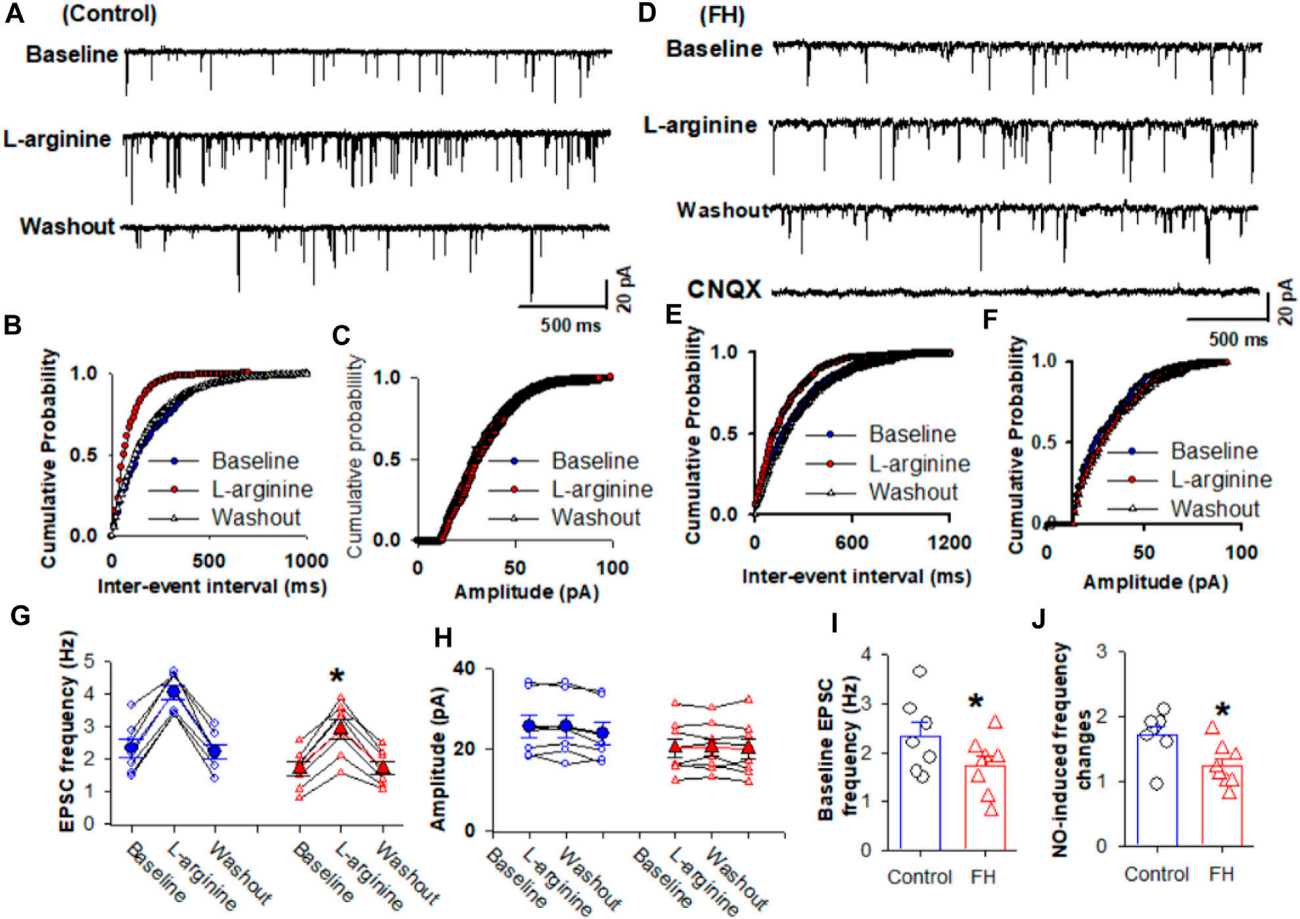
L-arginine-induced increase in miniature excitatory postsynaptic currents (mEPSCs) was reduced in fructose-fed rats. **(A)** Representative tracings show that the application of L-arginine (100 µM) increased the frequency of glutamatergic mEPSCs in CVLM-projecting NTS neurons in vehicle-treated rats. **(B,C)** Cumulative probability plot analysis of mEPSCs of the same neurons showing the distribution of the interevent interval **(B)** and peak amplitude **(C)** during control, L-arginine application, and washout. L-arginine shifted the distribution curves of the frequency of mEPSCs to the left (*p* <0.05; Kolmogorov–Smirnov test) without changing the distribution of their amplitude. **(D–F)** Representative tracings **(D)** and the cumulative probability plot analysis of the interevent interval **(B)** and peak amplitude **(C)**, which show that the application of L-arginine (100 µM) increased the frequency of mEPSCs without affecting their amplitude in CVLM-projecting NTS neurons in fructose-fed rats. L-arginine shifted the distribution curves of the frequency of mEPSCs to the left (*p* <0.05; Kolmogorov–Smirnov test) without changing the distribution of their amplitude. It should be noted that CNQX completely eliminated mEPSCs. **(G,H)** Summary data show the effect of 100 μM L-arginine on the frequency **(G)** and amplitude **(I)** of mEPSCs of 8 labeled PVN neurons. **(I,J)** Summary data show that the baseline frequency **(I)** of L-arginine-induced increases in the frequency **(J)** of mEPSCs in CVLM-projecting NTS neurons in fructose-fed rats was lower than that in vehicle-treated rats. Data are presented as the mean ± SEM (*p <0.05 compared with the control; Kruskal–Wallis ANOVA, followed by Dunn’s *post hoc* test). FH, fructose.

Since synaptic glutamate release is involved in the NO-induced increase in the firing activity of NTS neurons, we determined the effect of fructose feeding on L-arginine-glutamatergic synaptic inputs to the CVLM-projecting NTS neurons. The baseline frequency of mEPSCs was significantly lower in fructose-fed rats than that in vehicle-treated rats (Figures 3A, B, I). We found that L-arginine increased the frequency of mEPSCs recorded from the labeled NTS neurons in both fructose- and vehicle-treated rats (*p* <0.05; *n* = 8; Figures 3A–G). However, L-arginine did not alter the amplitude of mEPSCs. L-arginine-induced increases in the frequency were significantly reduced in fructose-fed rats compared with vehicle-treated rats (Figure 3J).

### 3.3 Fructose feeding reduces the NO-induced increase in evoked glutamatergic synaptic inputs to NTS neurons

**FIGURE 4:**
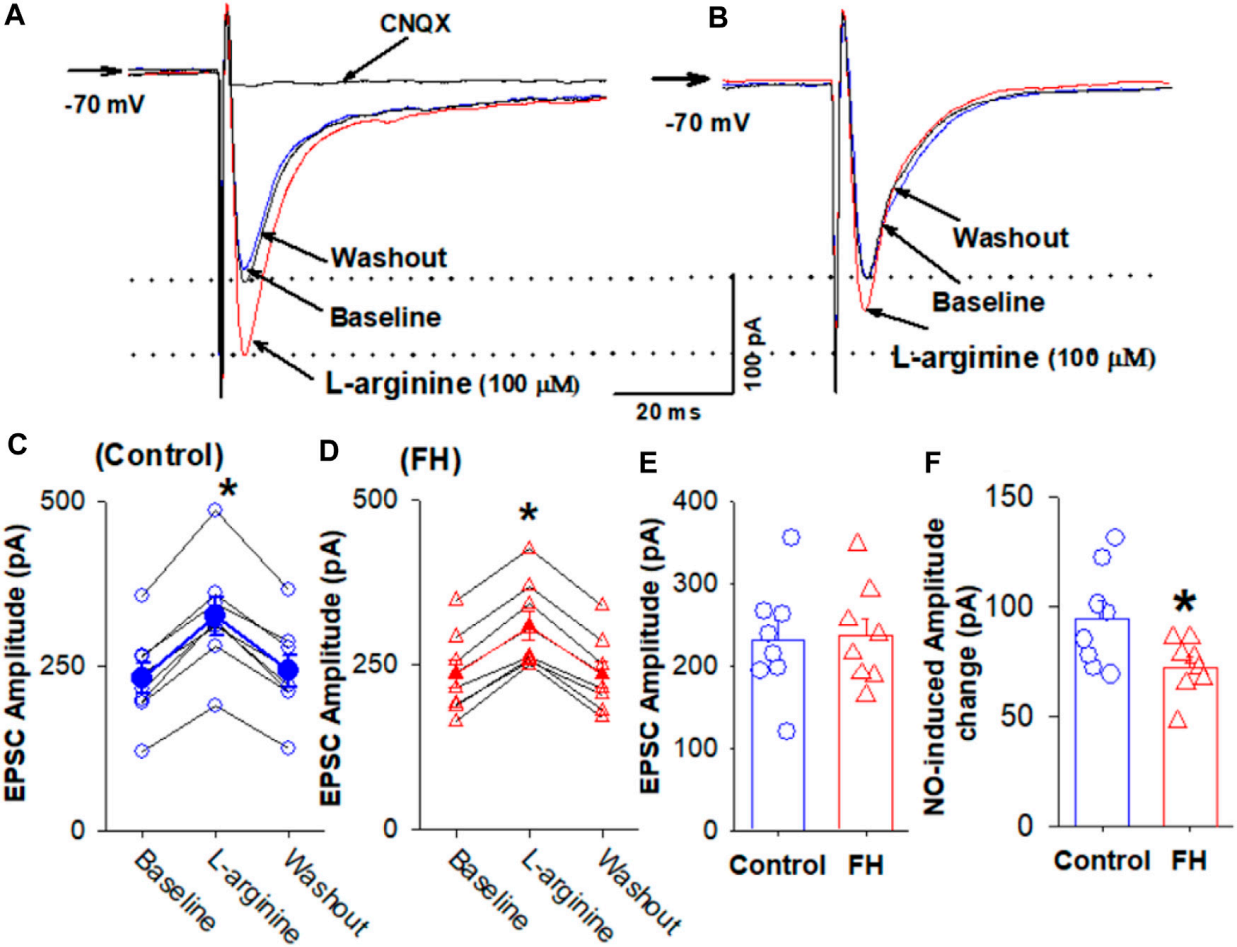
Effect of L-arginine on the evoked EPSCs in labeled CVLM-projecting NTS neurons in fructose- and vehicle-fed rats. **(A–D)** Representative raw tracings **(A,B)** and summary data **(C,D)** of evoked EPSCs show that 100 μM L-arginine increases the amplitude of evoked EPSCs; this effect was significantly smaller in fructose-fed rats than that in vehicle-treated rats. **(E,F)** Summary data show that the baseline amplitude **(E)** and L-arginine-induced increases in the amplitude **(F)** of evoked EPSCs in CVLM-projecting NTS neurons in fructose-fed rats were lower than those in vehicle-treated rats. Data are presented as the mean ± SEM (*p <0.05 compared with the control; ANOVA, followed by Dunn’s *post hoc* test). Traces are averages of 10 consecutive responses. FH, fructose.

Furthermore, we determined the effect of L-arginine on evoked EPSCs in CVLM-projecting NTS neurons in fructose-fed rats. The eEPSCs were recorded in the presence of 1 μM gabazine and 5 µM strychnine at a holding potential of −70 mV (Dias et al., 2003; Chen and Bonham, 2005). The eEPSCs were elicited by the electrical stimulation of the TS ipsilateral to the recording neurons (Li and Yang, 2007). L-arginine (100 µM) increased the peak amplitude of eEPSCs from 231.4 ± 24.2 to 325.7 ± 29.5 pA (*p* <0.05) in 8 labeled NTS neurons (Figure 4). In fructose-fed rats, the bath application of 100 μM L-arginine increased the peak amplitude of eEPSCs from 182.1 ± 9.0 to 257.1 ± 13.2 pA (*p* <0.05) in another group labeled as NTS neurons. The magnitude of the L-arginine-induced increase in the amplitude of eEPSCs was lesser in fructose-fed rats than that in vehicle-treated rats (Figure 4).

**FIGURE 5:**
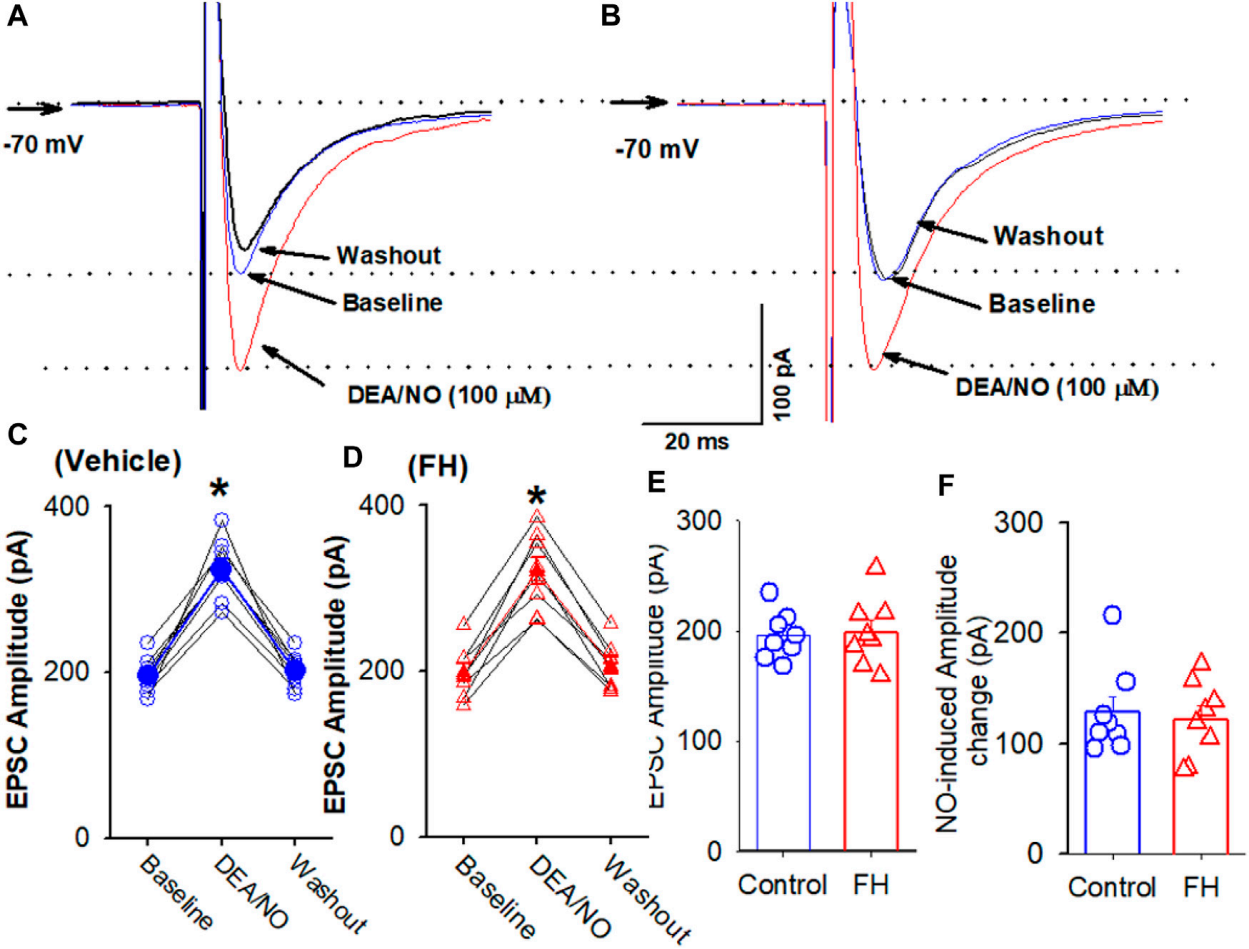
L-arginine produces similar increases in the amplitude of evoked EPSC CVLM-projecting NTS neurons in fructose- and vehicle-fed rats. **(A–D)** Representative raw tracings **(A,B)** and summary data **(C,D)** of evoked EPSCs show the effect of 100 μM L-arginine on the amplitude of evoked EPSCs in fructose-fed rats and vehicle-treated rats. **(E,F)** Summary data show the baseline amplitude **(E)** and L-arginine-induced increases in the amplitude **(F)** of evoked EPSCs in CVLM-projecting NTS neurons in fructose-fed rats and vehicle-treated rats. Data are presented as the mean ± SEM (*p <0.05 compared with the control; ANOVA, followed by Dunn’s *post hoc* test). Traces are averages of 10 consecutive responses. FH, fructose.

Because L-arginine produced lower increase in the amplitude of eEPSCs in the labeled NTS neurons in fructose-fed rats, we tested if this effect was due to decreased NO synthesis or the downregulation of NO signaling in labeled NTS neurons in fructose-fed rats. The bath application of NO donor DEA/NO, which releases NO directly, is independent of the NO synthesis. DEA/NO (100 µM) increased the amplitude of eEPSCs from 195.7 ± 7.5 to 324.5 ± 12.8 pA (*p* <0.05) in 8 labeled NTS neurons in fructose-fed rats, while in vehicle-treated rats, DEA/NO increased the amplitude of eEPSCs from 198.5 ± 10.8 to 320.3 ± 16.2 pA (*p* <0.05) in 8 labeled NTS neurons (Figure 5). The magnitude of the DEA/NO-induced increase in eEPSCs was similar in vehicle-treated and fructose-fed rats (Figure 5). These data suggested that the reduction in the L-arginine-induced increase in glutamatergic synaptic inputs results from a decrease in NO production rather than changes in the NO signaling pathways.

### 3.4 Fructose feeding reduces the NO-induced depressor response and sympathoinhibition

**FIGURE 6:**
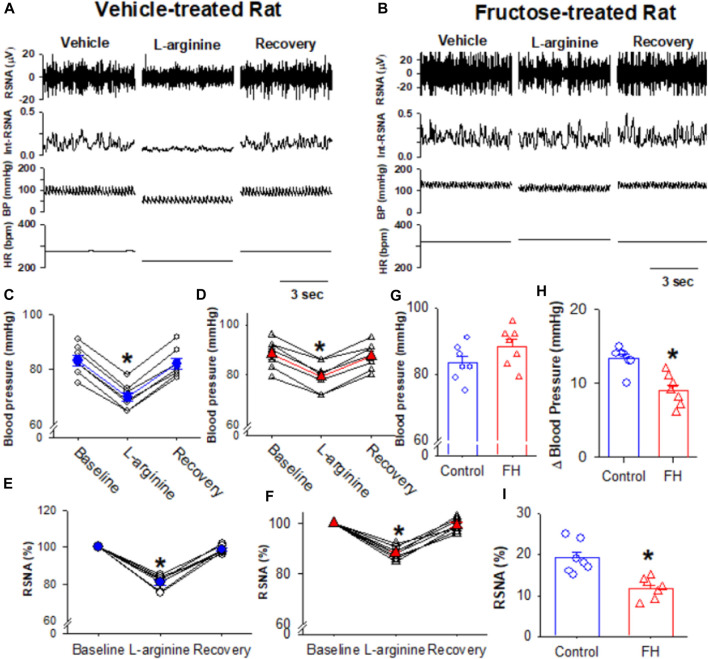
Microinjection of L-arginine into the NTS produced a lesser inhibition of the RSNA, blood pressure, and heart rate in fructose-fed rats than that in vehicle-treated rats. **(A,B)** Representative recordings show the effect of the bilateral microinjection of L-arginine (100 μM; 100 nL) into the NTS on the mean blood pressure and the RSNA in fructose-fed **(A)** and vehicle-treated rats **(B)**. **(C–F)** Summary data show the mean blood pressure **(C,D)** and the RSNA **(E,F)** in response to the microinjection of L-arginine into the NTS in fructose-fed **(D,F)** and vehicle-treated rats **(C,E)**. **(G)** Summary data show that the baseline blood pressure is higher in fructose-fed rats than that in vehicle-treated rats. **(H,I)** Summary data show that the L-arginine-induced reduction in blood pressure **(H)** and RSNA **(I)** in fructose-fed rats were smaller than those in vehicle-treated rats. Data are presented as the mean ± SEM. One-way ANOVA with the Bonferroni’s *post hoc* test was used to determine the differences between groups (*n* = 7 rats in each group). *p <0.05 compared with the respective baseline in each group. FH, fructose.

NO plays an important role in the regulation of the sympathetic nerve activity, arterial blood pressure, and heart rate in the NTS (Harada et al., 1993; Tseng et al., 1996; Matsumura et al., 1998). We found that the microinjection of the NO precursor L-arginine (10 nmol/50 nL) decreased the RSNA and blood pressure in both fructose- and vehicle-fed rats (Figures 6A–F). The L-arginine-induced decrease in the RSNA and blood pressure was significantly smaller in fructose-fed rats (Figures 6H, I). Since NO synthesis is required for L-arginine to produce NO, the reduced effect of L-arginine on the RSNA and blood pressure in fructose-fed rats suggested that endogenous NO production was decreased in the NTS in fructose-fed rats.

## 4 Discussion

This is the first study demonstrating that NO produces a less reduction in the blood pressure and sympathetic outflow in fructose-fed rats. It has been shown that people with metabolic syndrome are at an increased risk of developing type II diabetes and cardiovascular disease. The metabolic defects in fructose-fed rats resemble those in human metabolic syndrome, and fructose-fed rats are used as an animal model to study the inter-relationship between the metabolic defects and autonomic function without the confounding factors of obesity, diabetes, and hypertension. We found that fructose feeding reduces the NO precursor L-arginine-induced excitatory effect on NTS neurons. Furthermore, fructose feeding reduces the L-arginine-induced increase in the presynaptic spontaneous glutamatergic synaptic inputs to NTS neurons, while NO donor DEA/NO produces an increase in the glutamatergic synaptic inputs in fructose-fed rats similar to that in vehicle-treated rats. In addition, fructose feeding reduces the NO-induced depressor response and sympathoinhibition. These data suggested that fructose feeding reduced NO production and, thus, the subsequent NO-induced glutamate release in the NTS and depressor response. The findings of this study provide new insights into the central mechanisms involved in the neural control of cardiovascular and autonomic functions in the NTS in metabolic syndrome.

NO in the NTS plays a critical role in regulating autonomic nervous activity and cardiovascular reflexes (Tseng et al., 1996; Matsumura et al., 1998; Dias et al., 2005). The first-order neurons mediating the baroreflex, located at the NTS, receive afferents from baroreceptor neurons in the nodose ganglia (Aicher et al., 1999; Aicher et al., 2000). These NTS neurons innervate GABAergic neurons in the CVLM, which project to the sympathetic premotor neurons in the RVLM (Talman et al., 1980; Stornetta et al., 2016). This study assessed the NTS neurons projecting to the CVLM, which is labeled by the injection of a retrograde tracer into the CVLM (Li and Yang, 2007). In slice preparations, the labeled NTS neurons may lose some connection. Furthermore, the response of the neurons may differ between *in vitro* and *in vivo* conditions. Another intrinsic limitation of the slice recording technique is that only neurons relatively close to the surface of the tissue slice can be clearly visualized. We did not select damaged neurons (e.g., those with somatic swelling or missing or cut dendritic trees) or weakly labeled neurons for recordings.

It has been shown that the NO-induced depressor response in the NTS is mediated by its effect on the synaptic glutamate release (Lo et al., 1997; Lin et al., 1999). Furthermore, the NO donor increases the glutamatergic synaptic inputs in the NTS through the sGC-cGMP signaling pathway (Wang et al., 2007). Furthermore, nNOS and glutamate immunoreactivities are colocalized in the neuronal soma and fibers in the NTS (Lin L. H. et al., 2000). NO synthesis is impaired in insulin-resistant animals and patients (Sartori and Scherrer, 1999; Scherrer and Sartori, 2000). We speculate that NO-induced increases in the firing activity and glutamatergic synaptic inputs to autonomic NTS neurons are attenuated in fructose-fed rats. In this study, we found that the application of the NO precursor L-arginine increased the firing activity of CVLM-projecting NTS neurons, which are considered to convey baroreflex signaling from the NTS to the CVLM. The NO-induced increase in the firing rate of these neurons is consistent with the depressor response to the microinjection of NO into the NTS. We further determined the role of glutamatergic synaptic inputs in the effect of NO on the excitability of autonomic NTS neurons. The blockade of ionotropic glutamate receptors with 1*–*2 mM kynurenic acid eliminated the NO-induced increase in the firing activity of NTS neurons. These data suggest that NO stimulates the NTS neurons through the facilitation of presynaptic glutamate release to autonomic NTS neurons.

Accumulating evidence suggests that endothelial NO synthesis is impaired in insulin-resistant animals and patients (Sartori and Scherrer, 1999; Scherrer and Sartori, 2000). In this study, we determine the effect of the microinjection of the NO precursor L-arginine into the NTS on the RSNA, arterial blood pressure, and heart rate in fructose-fed rats and found that the L-arginine-induced depressor effect was reduced in fructose-fed rats. Due to the different numbers of nerve fibers recorded in each animal, it is difficult to directly compare the absolute values of the basal sympathetic nerve activity between fructose-fed and vehicle-treated rats. Furthermore, the L-arginine-induced increase in the firing activity and glutamatergic synaptic inputs was also reduced in fructose-fed rats compared with vehicle-treated rats. Because NO is synthesized by nNOS and eNOS in the NTS, the reduction in the L-arginine-induced depressor response is possibly due to a reduction in NO synthesis in the NTS. Our finding that the NO donor DEA/NO induced a similar increase in the evoked EPSCs in the NTS neurons in fructose-fed and vehicle-treated rats supports the notion that NO synthesis is reduced in the NTS in fructose-fed rats. Both eNOS and nNOS are expressed in the cell soma and nerve terminals in the NTS (Vincent and Kimura, 1992; Ruggiero et al., 1996; Takagishi et al., 2014). The findings of this study provide functional evidence that fructose treatment reduces the expression levels of eNOS and/or nNOS in the NTS, thereby reducing NO production, which is responsible for the decrease in glutamatergic synaptic inputs to the NTS neurons. However, this study did not measure the eNOS or nNOS expression levels in the NTS.

Another striking finding of our study is that the baseline firing activity and frequency of miniature glutamatergic EPSCs are lower in fructose-fed rats than those in vehicle-treated rats. The low-glutamatergic EPSCs may be involved in the low baseline firing activity in fructose-fed rats. Although the precise reasons that cause the low baseline firing activity and frequency of miniature glutamatergic EPSCs in fructose-fed rats are not clear, the reduction in NO production plays a critical role in the reduced baseline firing activity and frequency of glutamatergic EPSCs in fructose-fed rats. Because NO promotes glutamatergic synaptic inputs to NTS neurons, reduced endogenous NO produces less enhancement of glutamatergic synaptic inputs to support the tonic firing activity of NTS neurons in fructose-fed rats. In our study, we did not distinguish the source of glutamatergic synaptic inputs to CVLM-projecting NTS neurons since we recorded the miniature EPSCs, which represent glutamate synaptic inputs from all sources. However, we found that NO reduces evoked glutamatergic EPSCs by stimulating the TS, which contains the afferents from baroreceptors. Thus, we predict that the baroreflex may be reduced in fructose-fed rats due to a reduction in NO production. This reduced NO production in the NTS may contribute to the high blood pressure observed in fructose-fed rats.

NO in the NTS may act on other targets such as neurotransmission and ion channels in addition to enhancing glutamate synaptic inputs. Thus, reduced NO production in the NTS in response to fructose feeding contributes to high blood pressure through other pathways and mechanisms. For example, NO may alter GABAergic synaptic inputs to NTS neurons, which was not assessed in this study. In addition, NO may change ion channel activity, such as Ca^2+^ channels and K^+^ channels. It is not clear whether fructose feeding induces similar NO changes in other brain regions involved in regulating cardiovascular functions, such as blood pressure and autonomic nervous activity. This study determined the synaptic plasticity of neurons in the medial NTS subdivision project to the CVLM in fructose-fed rats. It is possible that other subdivisions of the NTS connected to the RVLM or PVN are also involved in fructose-induced metabolic syndrome. However, to the best of our knowledge, no available data support the notion that projections from the NTS to the RVLM or the NTS to the PVN are involved in fructose-induced metabolic syndrome. Future studies are warranted to elucidate the mechanisms involved in fructose-induced hypertension. Another limitation to this study is that only male rats were used. Gender is one of the crucial factors in the pathophysiological process in hypertension and diabetes. In this regard, women are less likely to suffer from cardiovascular diseases due to their high estrogen level (Ciarambino et al., 2022; Regensteiner and Reusch, 2022). It has been shown that estrogen expression levels are different in the NTS (Fan et al., 2010), suggesting gender differences in the NTS mechanism of fructose-induced metabolic syndrome.

## 5 Conclusion

In this study, fructose-induced metabolic syndrome and the excitatory effect of the NO precursor, L-arginine, on the firing activity and glutamatergic synaptic inputs of NTS neurons were found. However, the effect of NO donor DEA/NO on glutamatergic synaptic inputs did not change in NTS neurons. In addition, the NO-induced depressor response and sympathoinhibition were attenuated in fructose-induced metabolic syndrome. The findings from this study provide insights into a novel mechanism involved in the neural control of cardiovascular functions in metabolic syndrome.

## Data Availability

The original contributions presented in the study are included in the article/Supplementary Material; further inquiries can be directed to the corresponding authors.

## Abbreviations

ABP, arterial blood pressure; AMPA, α-amino-3-hydroxy-4-isoxazolepropionic acid; CVLM, caudal ventrolateral medulla; FH, fructose; eEPSCs, evoked excitatory postsynaptic currents; eIPSCs, evoked inhibitory postsynaptic currents; NOS, nitric oxide synthase; NMDA, N-methyl-D-aspirate; NO, nitric oxide; NTS, nucleus tractus solitarius; RSNA, renal sympathetic nerve activity; RVLM, rostral ventrolateral medulla.
